# Sudden cardiac death

**DOI:** 10.1080/20961790.2019.1622062

**Published:** 2019-08-19

**Authors:** Joaquín S. Lucena

**Affiliations:** Forensic Pathology Service, Institute of Legal Medicine and Forensic Sciences, Seville, Spain

Cardiovascular disease represents the main cause of death in developed countries. Cardiovascular disease frequently may account for premature fatal outcomes, even in the apparently healthy young, appearing in the form of sudden and unexpected death [1].

Sudden death (SD) is defined according to commonly accepted criteria in witnessed cases as follows. SD is a natural death that occurs within 6 h of the beginning of symptoms in an apparently healthy subject or in one whose disease is not so severe that a fatal outcome would be expected [2]. In cases of unwitnessed death, which is a common situation in forensic practice, this definition requires that the deceased was last seen alive and functioning normally 24 h before being found dead [3].

In approximately 75%–80% of SD cases, the origin of SD is the cardiovascular system. This situation is referred to as sudden cardiac death (SCD). In the diagnosis of SCD, those SDs resulting from disorders affecting different cardiovascular structures in which integrity is essential for normal heart function (e.g. coronary arteries, myocardium, cardiac valves, conduction system, intrapericardial aorta and pulmonary artery) are included [4]. SCD remains a leading cause of mortality and is responsible for approximately 50% of all deaths from cardiovascular disease, which accounts for an estimated 15%–20% of all deaths in Western societies [5, 6]. In this setting, SCD is considered a major international public health problem.

SCD of the young in apparently good health, although infrequent, is a dramatic event with social and clinical implications, and great concern for bereaved families and the community. The rate of SCD in people aged <35 years is approximately one to two cases per 100 000 people per year [7, 8]. In this age frame, the main causes are myocardial diseases and ion channel disorders (channelopathies). Among adults aged >35 years, the absolute rate of SCD markedly increases with age and sex, mainly in men, with a rate that is threefold higher than in women. However, recent studies suggest that this disparity may be declining [9]. In prospective studies that used standardized definitions and multiple sources of surveillance for case ascertainment, SCD rates ranged from 40 to 100 per 100 000 in the adult general population [10], with the lowest rates in China [11]. In adults aged >35 years, particularly among white men, coronary artery disease (CAD) is generally accepted as the most common cardiac pathology that is responsible for approximately 70%–75% of SCDs [5].

However, even among the young, CAD is a common cause of SCD. A study on SCD was performed at the Forensic Pathology Department, Institute of Legal Medicine and Forensic Sciences of Seville (Spain) over 10 years (2004–2013). In this study, the main cause of SCD in people aged 1–35 years (155 cases) was CAD (20%), followed by inherited cardiomyopathies (17%), pulmonary embolism (11%) and myocarditis (10%). The SCD rate in this age group was 1.7 per 100 000 persons per year (data not published) (Table 1).

**Table 1.** Causes of sudden cardiac death in 155 cases of forensic autopsies in people aged 1–35 years in Seville (Spain) (2004–2013).

Cause of death	Number (%)
Coronary artery disease	31 (20)
No abnormalities or unspecific findings	20 (13)
Pulmonary embolism	17 (11)
Myocarditis	15 (10)
Idiopathic left ventricular hypertrophy	12 (8)
Arrhythmogenic cardiomyopathy	12 (8)
Hypertrophic cardiomyopathy	9 (6)
Dilated cardiomyopathy	5 (3)
Congenital anomalies of coronaries	6 (4)
Thoracic aortic dissection	5 (3)
Aortic valve disease	4 (2.5)
Conduction system disease	4 (2.5)
Congenital heart disease operated	3 (2)
Morbid obesity cardiomyopathy	3 (2)
Cor pulmonale	2 (1)
Non-ischemic myocardial fibrosis	2 (1)
Long QT syndrome	1 (0.5)
Others	4 (2.5)
Total	155 (100)

In nearly two-thirds of cases, SCD is the first clinical manifestation of an underlying disease in previously asymptomatic, apparently “healthy” subjects, or in the setting of known cardiac disease in the absence of risk predictor. Therefore, an autopsy represents the first, and only, opportunity to establish and register an accurate cause of death [4]. However, autopsy rates are generally low and widely vary across countries, with rates as low as 10% of all deaths within the USA [12] compared with 23.8% in Finland [13]. The protocols for the performance of autopsies in cases of suspected SCD also widely vary, even within regions of countries. These differences in autopsy rates and protocols likely contribute to some of the geographical differences in the reported incidence and causes of SCD.

The Association for European Cardiovascular Pathology (AECVP) has developed guidelines for autopsy investigation of SCD that were recently updated [4, 14]. These guidelines include a detailed protocol for examination of the heart and recommendations for selection of histological blocks and appropriate material for toxicology, microbiology, biochemistry and molecular investigation. With adoption of these guidelines throughout Europe, the standards of autopsy practice will be improved, allowing meaningful comparisons between different communities and regions. This will permit identification of emerging patterns of diseases causing SCD [4].

After an autopsy and ancillary investigations, SCD may be classified into the following two main groups:

- SCD with a structural cardiovascular disease (*mors cum materia*), and most of them are due to hereditary diseases as follows:
  - Cardiomyopathies: dilated, hypertrophic, and arrhythmogenic.
  - Coronary artery disease.
  - Myocarditis.
  - Valve disease.
  - Congenital anomalies of coronaries.
  - Infiltrative diseases: sarcoidosis and amyloidosis.
  - Thoracic aortic dissection and aneurysms.
  - Pulmonary embolism.
- SCD with a structurally normal heart (*mors sine materia*), also known as sudden arrhythmic death syndrome by clinicians. This type of SCD might be due to the following inherited ion channel disorders [15–18]:
  - Long and short QT syndrome.
  - Brugada syndrome.
  - Catecholaminergic polymorphic ventricular tachycardia.
  - Early repolarization syndrome.

On the basis of the above-mentioned information, autopsy investigation of SCD should always include sampling for genetic testing to search for inherited arrhythmogenic disorders, as recommended in the recent guidelines by the AECVP [4]. Especially in SCD in young victims, genetic screening represents an important tool to support forensic investigation, and facilitates adequate risk stratification and genetic counselling [19].

Currently, we are witnessing a renaissance in cardiovascular pathology because of forensic pathology. The rapid and unexpected onset of SCD, which appears in an apparently healthy person, requires a forensic autopsy in the majority of countries. The aim of this autopsy is to exclude a violent mechanism and to determine the cause and manner of death. In Europe, autopsies in SCD must be performed following the Recommendations on the Harmonisation of Medico-Legal Autopsy Rules produced by the Committee of Ministers of the Council of Europe [20].

A total of 12.5% of naturally occurring deaths in Spain are estimated to be SD, which implies between 10 000 and 15 000 cases per year. Additionally, more than one half of medico-legal autopsies that are performed in the Department of Forensic Pathology are cases of natural deaths, and among them, SCDs constitute the most numerous group (75%). In this regard, forensic registries are a reliable source for assessing the impact of this problem in a given population [1].

Furthermore, autopsies play a pivotal role in identifying families at risk. The pathologist’s job is essential in counselling to refer first degree family members to cardiological screening and to perform postmortem genetic testing. The AECVP, as well as national cardiological societies, such us the Working Group of Familial Cardiomyopathies from the Spanish Society of Cardiology, recommend development of regional multidisciplinary networks of cardiologists, geneticists and pathologists. Their role will be to facilitate identification of index cases with a genetic basis, to screen appropriate family members, and ensure that appropriate preventive strategies are implemented [4, 21]. When confronted with an SCD, we should consider that we are not dealing with a single case, but with the entire family.

In this special issue on SCD, a relevant group of forensic pathologists, cardiovascular pathologists and cardiologists, who are all active members of the AECVP, have joined forces to present and update vision of this still complex topic. The main purpose of this issue is to assemble the knowledge and experience of different medical specialties, and different world regions for improving the diagnosis of SCD, gain a better understanding of risk factors and underlying conditions, and establish preventive strategies.

Joaquín S. Lucena *Forensic Pathology Service, Institute of Legal Medicine and Forensic Sciences, Seville, Spain* President of the Association for European Cardiovascular Pathology (AECVP) (aecvp.com) joaquin.lucena@gmail.com
